# The mycoparasitic fungus *Clonostachys rosea* responds with both common and specific gene expression during interspecific interactions with fungal prey

**DOI:** 10.1111/eva.12609

**Published:** 2018-03-14

**Authors:** Kristiina Nygren, Mukesh Dubey, Antonio Zapparata, Mudassir Iqbal, Georgios D. Tzelepis, Mikael Brandström Durling, Dan Funck Jensen, Magnus Karlsson

**Affiliations:** ^1^ Department of Forest Mycology and Plant Pathology Uppsala Biocenter Swedish University of Agricultural Sciences Uppsala Sweden; ^2^ Department of Agriculture, Food and Environment University of Pisa Pisa Italy; ^3^ Department of Plant Biology Uppsala Biocenter Linnean Centre for Plant Biology Swedish University of Agricultural Sciences Uppsala Sweden

**Keywords:** biological control, *Clonostachys rosea*, gene deletion, membrane transporter, mycoparasitism, transcriptome analysis

## Abstract

*Clonostachys rosea* is a necrotrophic mycoparasitic fungus, used for biological control of plant pathogenic fungi. A better understanding of the underlying mechanisms resulting in successful biocontrol is important for knowledge‐based improvements of the application and use of biocontrol in agricultural production systems. Transcriptomic analyses revealed that *C. rosea* responded with both common and specific gene expression during interactions with the fungal prey species *Botrytis cinerea* and *Fusarium graminearum*. Genes predicted to encode proteins involved in membrane transport, biosynthesis of secondary metabolites and carbohydrate‐active enzymes were induced during the mycoparasitic attack. Predicted major facilitator superfamily (MFS) transporters constituted 54% of the induced genes, and detailed phylogenetic and evolutionary analyses showed that a majority of these genes belonged to MFS gene families evolving under selection for increased paralog numbers, with predicted functions in drug resistance and transport of carbohydrates and small organic compounds. Sequence analysis of MFS transporters from family 2.A.1.3.65 identified rapidly evolving loop regions forming the entry to the transport tunnel, indicating changes in substrate specificity as a target for selection. Deletion of the MFS transporter gene *mfs464* resulted in mutants with increased growth inhibitory activity against *F. graminearum*, providing evidence for a function in interspecific fungal interactions. In summary, we show that the mycoparasite *C. rosea* can distinguish between fungal prey species and modulate its transcriptomic responses accordingly. Gene expression data emphasize the importance of secondary metabolites in mycoparasitic interactions.

## 1 INTRODUCTION

Chemical pesticides play an important role for maintaining high yields in agricultural productions systems in the world. However, the extensive use of agricultural chemicals has led to concerns about unwanted, negative effects on the environment and human health. One approach to minimize the amount of used pesticides is integrated pest management, which promotes the integrative use of preventive cultural practices, disease‐resistant plant cultivars and mechanical and biological control of pathogen populations. Biological control can also be used in organic agriculture, where chemical pesticides are banned. The use of biological control of plant pathogens by microbial antagonists can therefore be expected to increase, and to become standard procedure, in future agricultural and horticultural production.

Certain species of opportunistic, mycoparasitic fungi from the genera *Clonostachys* and *Trichoderma* are currently used as biological control agents to control plant pathogenic fungi. *Clonostachys rosea* (Link: Fr.) Schroers, Samuels, Seifert & W. Gams, comb. nov. (Schroers, Samuels, Seifert, & Gams, 1999) is an ascomycete fungus that is reported to control diseases caused by a wide range of plant pathogenic fungi, including *Alternaria* spp. (Jensen, Knudsen, Madsen, & Jensen, 2004), *Bipolaris sorokiniana* (Jensen, Knudsen, & Jensen, 2002), *Botrytis cinerea* (Sutton et al., 1997), *Fusarium culmorum* (Jensen, Knudsen, & Jensen, 2000), *Fusarium graminearum* (Xue et al., 2009) and *Sclerotinia sclerotiorum* (Rodriguez, Cabrera, Gozzo, Eberlin, & Godeas, 2011). Several biocontrol mechanisms are reported in *C. rosea*, including direct parasitism of pathogenic fungi (Li, Huang, Kokko, & Acharya, 2002; Yu & Sutton, 1997), antibiosis (Pachenari & Dix, 1980; Rodriguez et al., 2011), production of fungal cell wall degrading enzymes (Chatterton & Punja, 2009; Mamarabadi, Jensen, & Lubeck, 2008), induction of plant defence reactions (Lahlali & Peng, 2014; Roberti et al., 2008) and plant growth promotion (Roberti et al., 2008). Tolerance towards, and detoxification of, toxic metabolites produced by the fungal prey is also reported to be an important biocontrol trait in *C. rosea* (Dubey, Jensen, & Karlsson, 2014; Kosawang, Karlsson, Velez et al., 2014).

We recently sequenced the genome of *C. rosea* strain IK726 and performed a comparative genome analysis with *Trichoderma* and *Fusarium* species (Karlsson et al., 2015). Conspicuous features of the *C. rosea* gene content include a high number of genes encoding ATP‐binding cassette (ABC) and major facilitator superfamily (MFS) membrane transporters, polyketide synthase (PKS) and nonribosomal peptide synthetase (NRPS) secondary metabolite biosynthesis proteins, cytochrome P450 (CYP) and polysaccharide lyase family 1 (PL1) pectin lyases (Karlsson et al., 2015). The selection for high number of genes in these gene families suggests that their gene products may be involved in ecological niche adaptation (Wapinski, Pfeffer, Friedman, & Regev, 2007), such as mycoparasitism. Although *C. rosea* and *Trichoderma* spp. are necrotrophic mycoparasites with broad host range, it is not known if, or to what extent, they modulate their responses towards different fungal prey species. A better understanding of the mechanisms that determine the outcome of biocontrol interactions is necessary for science‐based improvements of biocontrol applications in agriculture.

The aim with the current work is to identify *C. rosea* genes that are specifically induced during interactions with the fungal pathogens *B. cinerea* or *F. graminearum*, and genes induced in response to both pathogens, using an RNA‐seq approach. We hypothesize that the transcriptomic response in *C. rosea* towards the two pathogens differs due to their intrinsic differences in cell wall composition, secondary metabolite spectra, etc. We further hypothesize that there will be an overlap between genes induced during fungal–fungal interactions and genes evolving under diversifying selection (in analogy with effectors in other parasite–host interactions). The results show that *C. rosea* responds with both common and specific transcriptional changes during interactions with *B. cinerea* and *F. graminearum*. Induction of genes putatively encoding drug resistance membrane transporters and proteins involved in biosynthesis of secondary metabolites emphasize the importance of secondary metabolites in mycoparasitic interactions.

## 2 MATERIALS AND METHODS

### 2.1 Cultivation conditions

*Clonostachys rosea* strain IK726 (WT) and mutants derived from it, *F. graminearum* strain PH‐1 and *B*. *cinerea* strain B05.10 were maintained on potato dextrose agar (PDA) medium (Oxoid, Cambridge, UK) at 25°C in darkness unless otherwise specified. Vogel's minimal (VM) medium (Vogel, 1956) with 0.3% glucose was used to grow fungi for transcriptome sequencing, while Czapek dox (CZ) medium (Sigma‐Aldrich, St. Louis, MO, USA) was used to grow fungi for reverse transcription quantitative PCR (RT‐qPCR) gene expression analyses, and phenotypic analyses of mutant strains.

For transcriptome sequencing during interactions, an agar plug of *C*. *rosea* mycelium and of the prey fungi *F*. *graminearum* or *B*. *cinerea* were inoculated at opposite sides of a 9‐cm‐diameter VM agar Petri plate (covered with a nylon membrane for easy harvest) and incubated at 25°C in darkness. Due to different mycelial growth rates, *C. rosea* was inoculated 5 days prior to the inoculation of *F*. *graminearum* or *B*. *cinerea*. The growing front (6 mm) of *C*. *rosea* mycelium was harvested together with the mycelial front (2 mm) of the prey fungi, 24 hr after hyphal contact between the fungi. Mycelium harvested from *C*. *rosea* confronted with itself at the same stage was used as the control treatment. Two biological replicates (different Petri plates) for each treatment were used.

For RT‐qPCR gene expression analysis, zearalenone (ZEA) was obtained from Sigma‐Aldrich and dissolved in methanol, while fungicides were obtained from BASF (Ludwigshafen, Germany [Cantus]), Syngenta (Basel, Switzerland [Amistar and Apron]) and Bayer AG (Leverkusen, Germany [Chipco Green and Teldor]) and dissolved in sterile distilled water. *Clonostachys rosea* was pregrown for 5 days in 20 ml liquid CZ medium, after which the growth medium was supplemented with ZEA to a final concentration of 10 μg/ml, Apron (mefenoxam) at 2 μg/ml, Amistar (azoxystrobin) at 7.5 μg/ml, Chipco Green (iprodione) at 250 μg/ml or Cantus (boscalid) at 2000 μg/ml. In the control treatments, ZEA and fungicides were replaced with an equal volume of methanol or sterile distilled water, respectively. Fungal mycelia were harvested 2 hr after the addition, washed in distilled water to remove traces of ZEA or fungicides, frozen in liquid nitrogen and stored at −80°C.

### 2.2 Nucleic acid isolation and manipulation

Genomic DNA was extracted from *C. rosea* as described previously (Nygren et al., 2008). RNA extraction was performed using the Qiagen RNeasy kit following the manufacturer's protocol (Qiagen, Hilden, Germany). After DNaseI (Fermentas, St. Leon‐Rot, Germany) treatment, five micrograms of total RNA was used for removal of ribosomal RNA using the Ribominus eukaryotic kit for RNA‐seq (Life Technologies, Carlsbad, CA, USA). For each sample, 500 nanograms of ribominus‐depleted RNA was reverse transcribed into cDNA using the SMARTer PCR cDNA synthesis kit following the manufacturer's protocol (Clontech, Mountain View, CA, USA). PCR amplifications were performed on cDNA for 15, 18, 21, 24 and 27 cycles to determine the optimum number of PCR cycles using the conditions: 95°C for 1 min, 95°C for 15 s, 64°C for 30 s and 65°C for 3 min on a GeneAMP PCR system 2700 (Applied Biosystems, Carlsbad, CA, USA). Running the PCR for 18 cycles was found to be optimal based on agarose gel electrophoresis (producing maximum amount of PCR products, but before entering the plateau phase of the reaction), and the PCR product was purified using a PCR purification kit (Fermentas, Waltham, MA, USA), analysed using a 2100 Bioanalyzer Instrument (Agilent Technologies, Santa Clara, CA, USA), quantified using a Qubit fluorometer (Life Technologies) and sent for paired‐end transcriptome sequencing at the SNP&SEQ Technology Platform, Uppsala, Sweden, using Illumina HiSeq2000 equipment. Read lengths were 107 and 144 bp, respectively, and the average insert sizes for the different samples were 305–340 bp.

### 2.3 Transcriptome data analysis

Quality filtration and adapter removal were performed using Cutadapt ver. 1.2.1 (Martin, 2011), minimum read length was set to 50 bp, and only reads still belonging to a complete pair were kept for further analysis. TopHat ver. 2.0.9 and Cufflinks ver. 2.1.1 packages (Trapnell et al., 2012) were used to map reads to the *C. rosea*,* B. cinerea* and *F. graminearum* reference genomes (Amselem et al., 2011; Cuomo et al., 2007; Karlsson et al., 2015), quantitate and test for differential gene expression. Parameter settings were kept at default, except for intron size adjustment. Fungi generally have short introns; therefore, minimal intron length was set to 5 bp and maximum intron length to 5,000 bp. Reads were analysed as unstranded, and read pair distance was adjusted for each sample. One mismatch was allowed in the analyses. The data were corrected for multiple testing by adjusting *p*‐values to a false discovery rate (FDR) of .05 using Cuffcompare in Cufflinks. Two of three conditions contained tissue from two species, *C. rosea* and either of *B. cinerea* and *F. graminearum*. Therefore, all reads were aligned to the three reference genomes and only the read pairs with a better alignment to the *C. rosea* genome than to either of the two other genomes were used in further analyses. To avoid biases in comparisons between conditions, all samples were filtered the same way. Expression levels were determined as reads per kilobase of transcript per million mapped reads.

### 2.4 Gene expression analysis with RT‐qPCR

RNA extraction was performed using the Qiagen RNeasy kit following the manufacturer's protocol (Qiagen). After DNaseI (Fermentas, St. Leon‐Rot, Germany) treatment, one microgram of total RNA was reverse transcribed in a total volume of 20 μl using Maxima first stand cDNA synthesis kit (Fermentas, St. Leon‐Rot, Germany). Transcript levels were quantified by RT‐qPCR using the SYBR Green PCR Master Mix (Fermentas, St. Leon‐Rot, Germany) and primer pairs listed in Appendix S1, in an iQ5 qPCR System (Bio‐Rad, Hercules, CA, USA) as described previously (Tzelepis, Melin, Jensen, Stenlid, & Karlsson, 2012). Melt curve analysis was performed after the qPCR reactions, to confirm that the signal was the result from a single product amplification. Relative expression levels for target genes in relation to actin (*act*), shown previously to be constitutively expressed (Kamou et al., 2016; Tzelepis, Dubey, Jensen, & Karlsson, 2015), were calculated from the Ct values by the $2^{-\Delta \Delta C_{t}}$ method (Livak & Schmittgen, 2001). Gene expression analysis was carried out in at least three biological replicates, each based on two technical replicates. Gene expression data were analysed by analysis of variance (ANOVA) using a general linear model approach implemented in Minitab ver. 18 (Minitab Inc., State College, PA, USA). Pairwise comparisons were made using the Fisher method at the 95% significance level.

### 2.5 MFS transporter gene family evolution

Seven hypocrealean fungi and *Neurospora crassa* were included in studying the evolutionary history of the MFS gene family. Whole‐genome nucleotide and protein sequences of the mycoparasitic fungi *C. rosea* (Karlsson et al., 2015), *T. atroviride*,* T. reesei* and *T. virens* (Kubicek et al., 2011; Martinez et al., 2008), and the plant pathogenic fungi *F. graminearum*,* F. solani* and *F. verticillioides* (Coleman et al., 2009; Cuomo et al., 2007; Ma et al., 2010), and the saprotrophic fungus *N. crassa* (Galagan et al., 2003) were retrieved from the National Center for Biotechnology Information (NCBI). ABC and MFS transporter genes were identified by BlastP analysis in an iterative process described previously (Karlsson et al., 2015), although the predicted *C. rosea* MFS transporters BN869_T00000646, BN869_T00007234 and BN869_T00002052 were additionally included as templates in the search. MFS transporters were classified into subgroups according to the Transporter Classification Database (TCDB, Saier et al., 2016).

Phylogenetic relationships were taken from Karlsson et al. (2015), while branch lengths were calculated based on a four‐gene alignment including actin, glyceraldehyde 3‐phosphate dehydrogenase, DNA‐directed RNA polymerase II subunit B and translation elongation factor 1 alpha. Coding gene sequences were retrieved from the respective genome sequences. Each gene was aligned individually using Clustal W (Larkin et al., 2007) in MEGA ver. 6 (Tamura, Stecher, Peterson, Filipski, & Kumar, 2013), concatenated and used to calculate branch lengths in MEGA ver. 6. The resulting species phylogeny was calibrated by setting the split between *T. reesei* and *T. virens* to 16 million years (de Man et al., 2016).

Major facilitator superfamily gene family evolution analysis was carried out on subgroups that contained ≥2 genes in at least one species and were present in ≥2 species. The program CAFE (Computational Analysis for Gene Family Evolution) ver. 3 (Han, Thomas, Lugo‐Martinez, & Hahn, 2013) was used to test whether gene family sizes were compatible with a stochastic birth‐and‐death model, to estimate gene family size in extinct species and to identify lineages with accelerated rates of gene gain or loss. Mutation rate (λ) was 0.0024 (Karlsson et al., 2015).

### 2.6 Phylogenetic analyses

Full‐length protein sequences were aligned by MUSCLE (Edgar, 2004), and phylogenetic trees were constructed using neighbour‐joining implemented in MEGA ver. 6. The JTT amino acid substitution model (Jones, Taylor, & Thornton, 1992) was used with uniform rates among sites and pairwise deletion of gaps. Statistical support for branches was assessed by 500 iterations of bootstrap resampling. Conserved protein modules and features were identified using the conserved domain database (CDD) (Marchler‐Bauer et al., 2017) at NCBI.

### 2.7 Analysis of molecular evolution

Regions of low amino acid conservation in MFS transporter alignments were identified by reverse conservation analysis (RCA), as described by Lee (2008). In short, Rate4Site ver. 2.01 was used to calculate the degree of conservation (S score, high scores correspond to low degree of conservation) for each amino acid position using the empirical Bayesian method (Mayrose, Graur, Ben‐Tal, & Pupko, 2004; Pupko, Bell, Mayrose, Glaser, & Ben‐Tal, 2002). A sliding‐window average (*n* = 7) of normalized S scores (mean was 0 and standard deviation was 1) was plotted in Excel (Microsoft; W mean score), and significant peaks were defined by values ≥1. Transmembrane helices were predicted using HMMTOP ver. 2 (Tusnady & Simon, 1998, 2001).

### 2.8 Construction of gene deletion vectors, transformation and mutant validation

Three fragment multisite gateway cloning system (Invitrogen, Carlsbad, CA, USA) was used to construct gene deletion vectors. The ~ 1 kb 5′‐flank and 3′‐flank regions of *mfs464*,* mfs602*,* fdo1* and *cyp1* were amplified from genomic DNA of *C*. *rosea* using gene specific primer pairs ups F/ups R and ds F/ds R, respectively, as indicated in Appendix S1. Gateway entry clones of the purified 5′‐flank and 3′‐flank PCR fragments were generated as described by the manufacturer (Invitrogen). The gateway entry clones for the hygromycin (*hph*) gene, constructed during our previous studies (Dubey, Broberg, Jensen, & Karlsson, 2013; Dubey, Ubhayasekera, Sandgren, Jensen, & Karlsson, 2012; Dubey, Broberg, Sooriyaarachchi et al., 2013), were used. The gateway LR recombination reaction was performed using entry plasmid of respective fragments and destination vector pPm43GW (Karimi, De Meyer, & Hilson, 2005) to generate the deletion vectors. *Agrobacterium tumefaciens*‐mediated transformation (ATMT) was performed based on a previous protocol for *C*. *rosea* (Utermark & Karlovsky, 2008). Putative transformants were selected on a plate containing hygromycin (200 μg/ml). Validation of homologous integration of the deletion cassettes in putative transformants was performed using a PCR screening approach with primer combinations targeting the *hph* gene (Hyg F/Hyg R) and sequences flanking the deletion cassettes (Appendices S1 and S2) as described previously (Dubey, Jensen, & Karlsson, 2016; Dubey et al., 2014). RT‐PCR analysis was conducted on WT and gene deletion strains using RevertAid premium reverse transcriptase (Fermentas, St. Leon‐Rot, Germany) and primer pairs specific for the respective genes (Appendix S1). The PCR‐positive transformants were tested for mitotic stability, and were purified by two rounds of single spore isolation (Dubey et al., 2014, 2016).

### 2.9 Phenotypic analyses of gene deletion mutants

For growth rate analysis, a 3‐mm‐diameter agar plug from the growing mycelial front was transferred to solid CZ medium, CZ medium containing the *Fusarium* mycotoxins ZEA (50 μg/ml) or deoxynivalenol (10 μg/ml); fungicides Cantus (boscalid [succinate dehydrogenase inhibitor] 2000 μg/ml), Amistar (azoxystrobin [electron transport chain inhibitor] 7.5 μg/ml), Chipco green (iprodione [inhibitor of DNA and RNA synthesis, and NADH cytochrome c reductase] 250 μg/ml), Apron (mefenoxam [inhibitor of RNA polymerases] 2 μg/ml) or Teldor (fenhexamid [inhibitor of sterol biosynthesis] 5000 μg/ml); inducers of oxidative stress paraquat (10 mM) or H_2_O_2_ (10 mM), cell wall stress SDS (0.05%) or caffeine (0.1%), osmotic stress sodium chloride (0.5 M) or sorbitol (1 M); ions calcium chloride (400 mM), potassium chloride (400 mM), magnesium chloride (400 mM), caesium chloride (20 mM), zinc chloride (20 mM) or lithium chloride (10 mM). In addition, growth rates were analysed under nutrient‐limited conditions at pH 4.0, pH 9.0 or pH 10.0. Limitation for carbon, nitrogen, magnesium, iron or potassium was induced by reducing the concentration of sucrose, sodium nitrate, magnesium sulphate, ferrous sulphate or potassium chloride by 1/10. Colony diameter was measured after 5 days of growth at 25°C in darkness. The concentration of chemicals used for phenotypic analysis in this study was based on our previously published results (Dubey et al., 2014, 2016) with the exception of ions. The appropriate concentration of ions was selected based on screening of *C*. *rosea* WT spores and mycelium on CZ plates amended with a series of different concentrations of the respective compounds. For determination of conidial germination, germ tube development and colony formation, WT and deletion strain conidia were inoculated (10^3^ conidia) on solid agar plates using the same concentration of chemical compounds described above with the exception of H_2_O_2_ (0.8 mM) and Teldor (fenhexamid 1000 μg/ml). Data for conidial germination and germ tube development were recorded daily using a Leica 165FC microscope (Wetzlar, Germany), while colony diameter was measured 5 days postinoculation. All phenotypic analyses were performed with at least three independent mutants (to avoid phenotypes resulting from ectopic insertions of the deletion cassette), and each based on at least three biological replicates unless otherwise specified.

Antagonistic behaviour of *C*. *rosea* WT and deletion strains against *F. graminearum* or *B. cinerea* was tested in five biological replicates using an in vitro plate confrontation assay and a culture filtrate assay, as described previously (Dubey et al., 2014, 2016). In addition, a liquid culture confrontation test was performed in five biological replicates, where conidia (10^5^ conidia) from WT and deletion strains were inoculated in 50 ml VM with 0.3% glucose in Erlenmeyer flasks and were allowed to grow for 24 hr at 25°C in darkness. Subsequently, an equal amount of *F*. *graminearum* spores were added to the flasks and further incubated for 3 days to allow the antagonistic interaction. All mycelia were harvested, washed with distilled water and used for DNA extraction. The mycelial biomass was estimated by measuring *C*. *rosea* and *F. graminearum* DNA with qPCR using primers specific to β‐tubulin (Dubey et al., 2014; Reischer, Lemmens, Farnleitner, Adler, & Mach, 2004). An in vivo bioassay using a sand seedling test for fusarium foot rot disease on barley was performed in five biological replicates, where each replicate included 15 plants, following the procedure described previously (Dubey et al., 2014, 2016).

## 3 RESULTS

### 3.1 Transcriptional response of *C. rosea* during interactions with *B. cinerea* and *F. graminearum*

Dual confrontation assays (*C. rosea* vs. *C. rosea*,* C. rosea* vs. *B. cinerea* and *C. rosea* vs. *F. graminearum*) were monitored using a stereo microscope on a daily basis, and mycelium from the interaction zone was harvested 24 hr after the first hyphal contacts were observed. This occurred 10 days after inoculation of *C. rosea* for all interactions. Illumina HiSeq paired‐end sequencing of cDNA generated from RNA from the interaction zones resulted in a total of 117 million read pairs, equivalent to 143.5 Gbp of sequence. After removal of low‐quality reads and reads originating from *B. cinerea* or *F. graminearum*, 36.8 million read pairs remained that were used for analysis and identification of differentially expressed genes. In total, 41 differentially expressed genes were identified, and functional classification showed that 61% were predicted to encode membrane transporters, 12% were predicted to encode proteins involved in biosynthesis of secondary metabolites, and 7% encoded carbohydrate‐active enzymes (Table 1). Six genes were induced in *C. rosea* during interactions with both *B. cinerea* and *F. graminearum*, while most genes were induced specifically towards either of the two different fungal prey species (Table 1).

**Table 1:** Differentially expressed genes in *Clonostachys rosea* during interactions with *Botrytis cinerea* or *Fusarium graminearum*

Protein ID	Gene name	Putative function	E‐value^a^	Expression level in CR^b^	Expression level in BC^b^	Expression level in FG^b^
BN869_T00005393		Hydantoinase/oxoprolinase	0	2.7	**16.5**	**16.5**
BN869_T00013543		2‐deoxy‐d‐gluconate 3‐dehydrogenase	1e‐160	3.6	**24.5**	**35.5**
BN869_T00013396		Uracil permease	0	5.3	**50.0**	**82.3**
BN869_T00010526	*mfs24*	Maltose permease (MFS)	0	14.4	**67.2**	**47.2**
BN869_T00005531	*mfs166*	Carboxylic acid transporter (MFS)	0	1.0	**7.1**	**10.2**
BN869_T00007347	*mfs104*	Glucose transporter (MFS)	0	29.5	**77.0**	**76.0**
BN869_T00006915		PL1 pectate lyase	5e‐165	28.4	101.9*	**4.0**
BN869_T00008662		Oxalate decarboxylase	0	0.6	20.7*	0.8
BN869_T00008498		Cytochrome P450	0	1.4	43.7*	3.7
BN869_T00004216		Oxalate decarboxylase	0	14.2	110.1*	7.9
BN869_T00009370		Cyclopentanone monooxygenase	0	50.8	199.7*	65.1
BN869_T00006567	*pks9*	Lovastatin diketide synthase	0	0.7	2.3*	0.3
BN869_T00012005	*nps13*	Nonribosomal peptide synthetase	0	1.6	3.0*	0.6
BN869_T00012311	*mfs293*	MFS transporter	0	1.8	16.4*	2.5
BN869_T00009923	*mfs249*	MFS transporter	2e‐107	7.5	36.9*	11.7
BN869_T00007301	*mfs44*	Sugar/quinate transporter (MFS)	0	16.8	**57.2**	23.0
BN869_T00006570	*abcC8*	ABC transporter	0	0.7	2.7*	0.4
BN869_T00008321		GH28 alpha‐L‐rhamnosidase	8e‐180	1.1	2.2	18.3*
BN869_T00006501	*chiC1*	GH18 killer toxin‐like chitinase	0	1.8	1.9	90.4*
BN869_T00000852	*cyp1*	Isotrichodermin C‐15 hydroxylase	0	2.7	0.9	35.4*
BN869_T00011161		Norsolorinic acid reductase	0	29.4	28.8	108.4*
BN869_T00006455	*mfs602*	MFS transporter	0	0.7	0.2	**485.3**
BN869_T00007234	*mfs464*	MFS transporter	0	0.9	0.9	709.9*
BN869_T00010393	*mfs49*	Quinate transporter (MFS)	0	1.0	1.1	23.0*
BN869_T00000606	*fdo1*	FAD‐dependent oxidoreductase	4e‐127	1.4	1.6	121.0*
BN869_T00009314	*mfs271*	Allantoate permease (MFS)	0	3.6	6.9	58.1*
BN869_T00002607	*mfs518*	MFS transporter	0	3.8	3.6	54.0*
BN869_T00005826		Potassium channel subunit beta	0	3.9	3.3	51.3*
BN869_T00008599	*mfs187*	MFS transporter	1e‐148	8.7	9.9	37.8*
BN869_T00007004	*mfs534*	MFS transporter	0	12.3	11.0	46.7*
BN869_T00009913	*mfs524*	Cycloheximide resistance protein (MFS)	0	12.9	6.5	75.9*
BN869_T00002418	*ptr1*	Peptide transporter	0	27.0	32.5	103.0*
BN869_T00010026	*abcG18*	ABC transporter	0	0.5	0.5	2.8*
BN869_T00011684	*mfs324*	Mononucleotide permease (MFS)	0	1.1	1.5	12.3*
BN869_T00005590	*mfs604*	MFS transporter	0	2.2	1.7	8.3*
BN869_T00006700	*mfs315*	Pantothenate transporter (MFS)	0	69.3	73.1	232.0*
BN869_T00012557	*mfs205*	Riboflavin transporter (MFS)	9e‐169	29.3	17.0	83.2*
BN869_T00008447	*mfs282*	MFS transporter	0	1.6	1.4	6.0*
BN869_T00013156	*mfs291*	Vitamin H transporter (MFS)	0	2.0	1.8	8.3*
BN869_T00000646	*mfs176*	MFS transporter	4e‐145	15.4	12.5	44.6*
BN869_T00011371	*mfs609*	Aminotriazole resistance protein (MFS)	0	45.4	43.7	109.5*

ABC, ATP‐binding cassette; FAD, flavin adenine dinucleotide; GH, glycoside hydrolase; MFS, major facilitator superfamily; PL, polysaccharide lyase.

a E‐value originates from BlastP analysis of the NCBI nonredundant database, or from the SMART protein analysis tool.

b Expression level was determined as reads per kilobase of transcript per million mapped reads values. Treatments are CR = expression level in treatment *C. rosea* vs. *C. rosea*, BC = expression level in treatment *C. rosea* vs. *B. cinerea*, FG = expression level in treatment *C. rosea* vs. *F. graminearum*. BC and FG values indicated in bold are significantly (FDR adjusted *p* ≤ .05) different from CR values in normal font. BC or FG values marked with an asterisk are significantly (FDR adjusted *p* ≤ .05) higher in the comparison between BC and FG treatments.

Among the 26 differentially expressed membrane transporter genes, 22 were identified as putative MFS transporters while only two were ABC transporters. The two ABC transporter genes were described previously as *abcC8* and *abcG18* (Karlsson et al., 2015) and predicted to encode members of the multidrug resistance‐associated protein (MRP, group C) and the pleiotropic drug resistance protein (PDR, group G) ABC transporter groups, respectively. Expression of *abcC8* was induced during interaction with *B. cinerea*, while *abcG18* was induced specifically against *F. graminearum* (Table 1). Three putative MFS transporter genes and one NCS1‐type family uracil transporter (BN869_T00013396) were induced against both pathogens compared with the *C. rosea* self‐interaction, while 3 and 16 MFS transporter genes were specifically induced against *B. cinerea* and *F. graminearum*, respectively. A putative membrane peptide transporter (BN869_T00002418 [*ptr1*]) was also induced 3.8‐fold against *F. graminearum* specifically. The highest fold‐change inductions were detected for two predicted MFS transporters (BN869_T00006455 [*mfs602*] and BN869_T00007234 [*mfs464*]) that were induced 693‐fold and 789‐fold, respectively, during interaction with *F. graminearum* (Table 1).

The PKS gene *pks9*, putatively encoding a reduced polyketide‐type PKS, and the NRPS gene *nps13* (Karlsson et al., 2015) were induced during confrontation with *B. cinerea* in comparison with *F. graminearum* although expression of *nps13* was not significantly different from the *C. rosea* self‐interaction control treatment. A predicted CYP (BN869_T00000852 [*cyp1*]) with similarity to an isotrichodermin C‐15 hydroxylase was induced specifically against *F. graminearum*. The killer toxin‐like chitinase gene *chiC1* (Tzelepis et al., 2015) was induced 50‐fold during interactions with *F. graminearum*, in comparison with the *B. cinerea* and *C. rosea* self‐interaction treatments. A predicted FAD‐dependent oxidoreductase (BN869_T00000606 [*fdo1*]) with similarity to a tetracycline resistance protein was induced 86‐fold against *F. graminearum*. A putative PL1 pectin lyase gene (BN869_T00006915) was induced during interaction with *B. cinerea*, but repressed during interaction with *F. graminearum* (Table 1).

### 3.2 Validation of differential expression by RT‐qPCR

**Figure 1:**
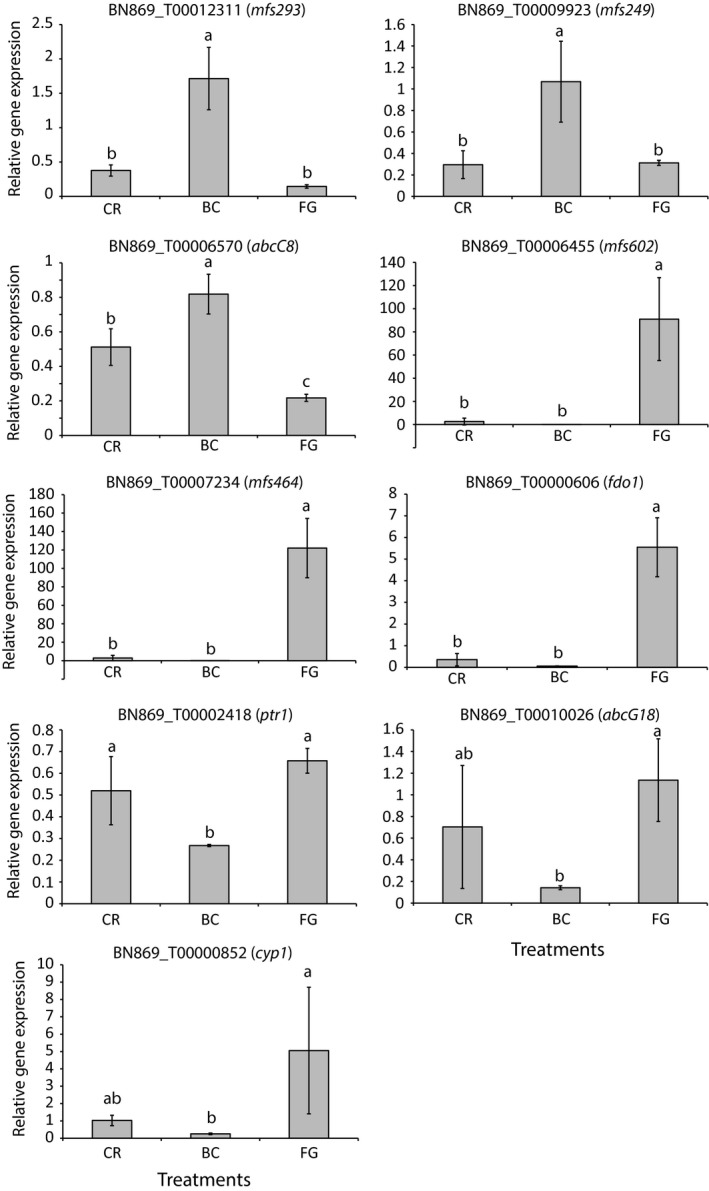
Expression analysis of *Clonostachys rosea* genes. Gene expression of *C. rosea* genes was measured with RT‐qPCR during interactions with *Botrytis cinerea* (BC) or *Fusarium graminearum* (FG) or during self‐interactions (CR). Relative gene expression was calculated using the $2^{-\Delta \Delta C_{t}}$ method, normalized by actin (*act*) expression. Error bars represent standard deviation based on three biological replicates. Different letters indicate statistically significant differences (*p* ≤ .05) based on the Fisher method

Validation of RNA‐seq gene expression data with RT‐qPCR was carried out for nine selected genes, including three genes specifically induced during interaction with *B. cinerea* and six genes specifically induced against *F. graminearum*. There was a high level of congruence between gene expression patterns measured with the two methods (Table 1, Figure 1). Four minor differences were detected; expression of *abcC8* was higher in the *C. rosea* vs. *C. rosea* treatment compared with the *F. graminearum* confrontation using RT‐qPCR but not with RNA‐seq, while expression of *ptr1*,* abcG18* and *cyp1* was higher in the *F. graminearum* confrontation compared with the *C. rosea* vs. *C. rosea* treatment using RNA‐seq but not with RT‐qPCR (Figure 1).

### 3.3 Evolution of the MFS transporter gene family in *C. rosea* and other hypocrealean fungi

The massive induction of membrane transporter genes in *C. rosea* during confrontations with fungal prey prompted us to investigate these gene families in more detail. The ABC transporter gene family in *C. rosea* was previously described in detail (Karlsson et al., 2015), but an iterative Blast approach identified an additional four genes resulting in a total number of 90 ABC transporter genes in the *C. rosea* genome. Based on a phylogenetic analysis (data not shown), these four additional ABC transporter genes were classified as members of subgroup E (BN869_T00001090) and subgroup F (BN869_T00005647, BN869_T00006720 and BN869_T00007586) according to the Kovalchuk and Driessen (2010) classification system, and named *abcE1*,* abcF3*,* abcF4* and *abcF5*, respectively.

Using an iterative Blast approach, we further identified a total of 634 MFS transporter genes in the genome of *C. rosea* (Appendix S3). The total number of MFS transporter genes in other hypocrealean fungal species ranged from 197 in *T. reesei* to 587 in *F. solani*, while *N. crassa* possessed 132 genes (Appendix S3). All predicted MFS transporters were classified into 124 different subgroups based on sequence similarity and predicted substrate transport preference, according to the TCDB (Saier et al., 2016). The phylogenetic relationship of *C. rosea* with seven other fungi with varying life strategies (Appendix S4) was used for analysis of MFS transporter gene family expansions and contractions with the program CAFE.

In total, 35 MFS gene families were identified to evolve nonrandomly (*p* ≤ .05) in at least one species (Appendix S5). In *C. rosea* specifically, 25 MFS gene families were identified to contain more genes than expected given genetic drift, thereby implying selection to maintain gene duplicates (Table 2). In contrast, no contracted MFS gene families were identified in *C. rosea*. Based on the predicted functions from TCDB, these 25 MFS gene families were divided into three broad categories; six families involved in drug resistance/secondary metabolite transport, eight families involved in small organic compound transport, and six families involved in carbohydrate transport (Table 2). In addition, one family (MFS 2.A.1.14.38) were predicted to transport sulphur‐compounds, one family (MFS 2.A.1.16.7) were predicted to transport siderophores, while three families lacked any described function. In comparison with the three mycoparasitic *Trichoderma* species, only two MFS gene families were identified as expanded in both *C. rosea* and *T. atroviride* (but not *T. virens* or *T. reesei*); MFS 2.A.1.3.65 (multidrug resistance) and MFS 2.A.1.3.68 (acid resistance and pH homoeostasis; Table 2). Three additional MFS gene families were identified as expanded in *T. atroviride* (MFS 2.A.1.2.6: multidrug resistance, MFS 2.A.1.2.35: caffeine resistance, MFS 2.A.1.3.32: drug resistance) but not in *T. virens* or *T. reesei*, while one MFS gene family was expanded in *T. virens* (MFS 2.A.1.2.79: toxin exporter) but not in *T. atroviride* or *T. reesei* (Appendix S5).

**Table 2:** Major facilitator superfamily (MFS) gene numbers in groups evolving nonrandomly^a^ in *C. rosea*

MFS classification^b^	MFS annotation^b^	CR	FS	FV	FG	TA	TV	TR	NC
MFS 2.A.1.1.7	Quinate: H+ symporter	**9**	5	8	10	3	4	3	*1*
MFS 2.A.1.1.11	α‐glucoside: H+ symporter	**27**	**31**	20	*16*	9	12	*6*	*1*
MFS 2.A.1.1.57	Monosaccharide:H+ symporter	**8**	**7**	4	*1*	2	2	1	4
MFS 2.A.1.1.68	Glucose transporter/sensor	**5**	**7**	5	*1*	0	0	0	2
MFS 2.A.1.1.83	Cellobiose/cellodextrin transporter	**24**	**33**	24	*19*	*5*	8	*3*	*4*
MFS 2.A.1.1.119	Galacturonic acid uptake porter	**19**	14	14	*7*	10	6	5	*4*
MFS 2.A.1.2.33	Unknown function	**17**	**25**	**24**	*11*	6	6	6	*3*
MFS 2.A.1.2.77	Drug resistance transporter	**18**	13	10	11	12	8	*6*	*5*
MFS 2.A.1.2.85	Peroxisomal phenylacetate transporter	**3**	**3**	1	0	*0*	1	0	0
MFS 2.A.1.2.86	Peroxisomal isopenicillin N importer	**24**	**21**	16	*9*	14	13	*8*	*4*
MFS 2.A.1.3.33	Multidrug resistance porter	**6**	3	*1*	3	1	2	1	*0*
MFS 2.A.1.3.47	Tri12 trichothecene efflux pump	**6**	4	**5**	2	0	0	0	2
MFS 2.A.1.3.65	Multidrug resistance transporter	**33**	**25**	21	*14*	**24**	19	*8*	*7*
MFS 2.A.1.3.68	Acid resistance and pH homoeostasis	**4**	**2**	0	0	**2**	1	0	0
MFS 2.A.1.13.4	Riboflavin uptake transporter	**16**	**16**	7	6	11	7	8	*1*
MFS 2.A.1.13.19	Unknown function	**20**	12	13	*9*	7	9	*6*	*4*
MFS 2.A.1.14.3	Tartrate porter	**20**	**12**	10	*8*	3	5	*2*	*4*
MFS 2.A.1.14.4	Dipeptide porter	**30**	**29**	16	12	9	11	*4*	*1*
MFS 2.A.1.14.8	Phthalate porter	**6**	1	**3**	2	0	0	0	*0*
MFS 2.A.1.14.11	Nicotinate permease	**93**	**51**	37	34	22	23	*16*	*13*
MFS 2.A.1.14.17	Pantothenate: H+ symporter	**19**	**18**	8	9	2	3	2	*2*
MFS 2.A.1.14.36	Thiamine transporter	**14**	8	9	7	9	9	*6*	*1*
MFS 2.A.1.14.37	Unknown function	**5**	**9**	2	1	1	0	*0*	2
MFS 2.A.1.14.38	Inorganic sulphur‐compound transporter	**11**	**16**	**13**	*6*	4	4	*2*	*3*
MFS 2.A.1.16.7	Ferri‐siderophore transporter	**15**	**13**	7	7	4	6	*3*	*1*

Species abbreviations: CR, *C. rosea*; FG, *F. graminearum*; FS, *F. solani*; FV, *F. verticillioides*; NC, *N. crassa*; TA, *T. atroviride*; TR, *T. reesei*; TV, *T. virens*.

a Gene numbers in bold indicates a significant (*p* ≤ .05) expansion, while gene numbers in italic indicates a significant (*p* ≤ .05) contraction of gene family size.

b MFS classification and annotation are based on BlastP analysis (E‐value ≤ 1e‐5) of the TCDB transporter classification database.

Nineteen of the 22 differentially expressed *C. rosea* MFS transporter genes belonged to expanded MFS gene families (Table 2), including five genes in gene family MFS 2.A.1.14.11 (nicotinate permease), three genes in gene family MFS 2.A.1.2.86 (peroxisomal isopenicillin N importer) and two genes each in gene families MFS 2.A.1.1.119 (galacturonic acid uptake) and MFS 2.A.1.14.17 (pantothenate uptake). The highly induced MFS transporter genes *mfs602* (BN869_T00006455) and *mfs464* (BN869_T00007234) belonged to the expanded MFS gene families 2.A.1.3.65 (multidrug resistance) and 2.A.1.2.33 (unknown function), respectively.

### 3.4 Phylogenetic relationships of selected MFS transporter gene families

**Figure 2:**
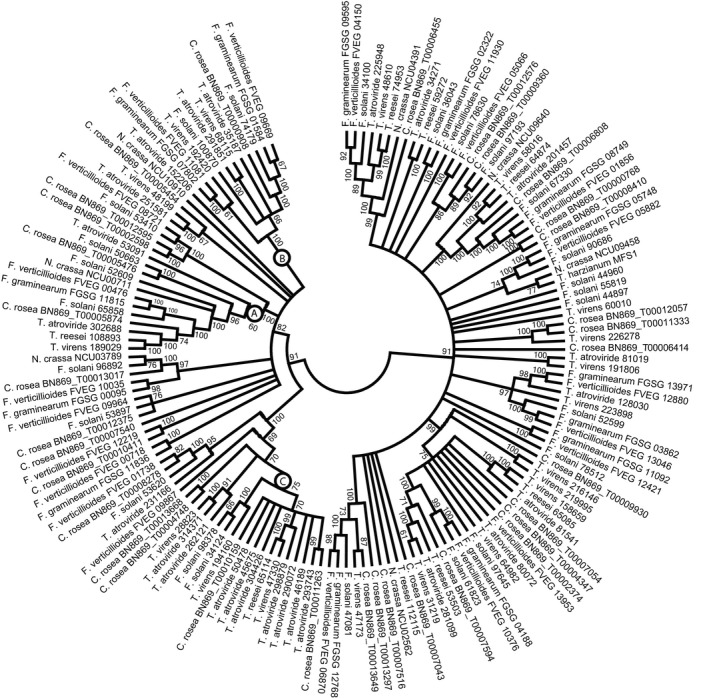
Phylogenetic relationships of MFS transporter family 2.A.1.3.65 among Hypocreales. Predicted amino acid sequences of MFS transporters were aligned by MUSCLE and used to construct a phylogenetic tree using the neighbour‐joining method in the MEGA6 software package. Bootstrap support (≥60%) values from 500 iterations are associated with nodes. Species name and protein ID are given for each MFS transporter

Guided by the high (≥693‐fold) induction of *mfs602* and *mfs464* during interactions with *F. graminearum*, but not against *B. cinerea*, we analysed the phylogenetic relationships of the expanded MFS transporter gene families 2.A.1.3.65 (multidrug resistance) and 2.A.1.2.33 (unknown function). The phylogenetic tree of the MFS 2.A.1.3.65 gene family displayed low resolution among the deeper branches, and incongruence with the species phylogeny in many subgroups (Figure 2), indicative of a birth‐and‐death evolutionary process followed by rapid sequence divergence. The 33 *C. rosea* paralogous genes were distributed over the phylogeny, rather than being overrepresented in a specific subgroup. The predicted *C. rosea* MFS602 protein clustered in a well‐supported (99% bootstrap support) subgroup together with single orthologs from *Fusarium* spp. and *Trichoderma* spp. (Figure 2). The analysis also showed that *C. rosea* MFS602 was not orthologous to the previously characterized family 2.A.1.3.65 *T*. cf. *harzianum* MFS1 transporter involved in trichodermin secretion and protection against mycotoxins and xenobiotics (Liu, Liu, & Wang, 2012).

The MFS 2.A.1.3.65 gene family was also expanded in *T. atroviride*, but not in *T. virens* or *T. reesei*. In contrast to *C. rosea*, the major expansion in *T. atroviride* was traced to a specific subgroup; seven *T. atroviride* paralogs clustered together with genes from the other mycoparasites *T. virens*,* T. reesei* and *C. rosea* (Figure 2). Pairwise DNA identity between the seven paralogs was between 46.8% and 59.1%. The seven paralogs were not localized in tandem repeats, but in four different scaffolds in the *T. atroviride* genome (data not shown). No association with predicted secondary metabolite clusters in *T. atroviride* was found for any of the seven MFS genes.

**Figure 3:**
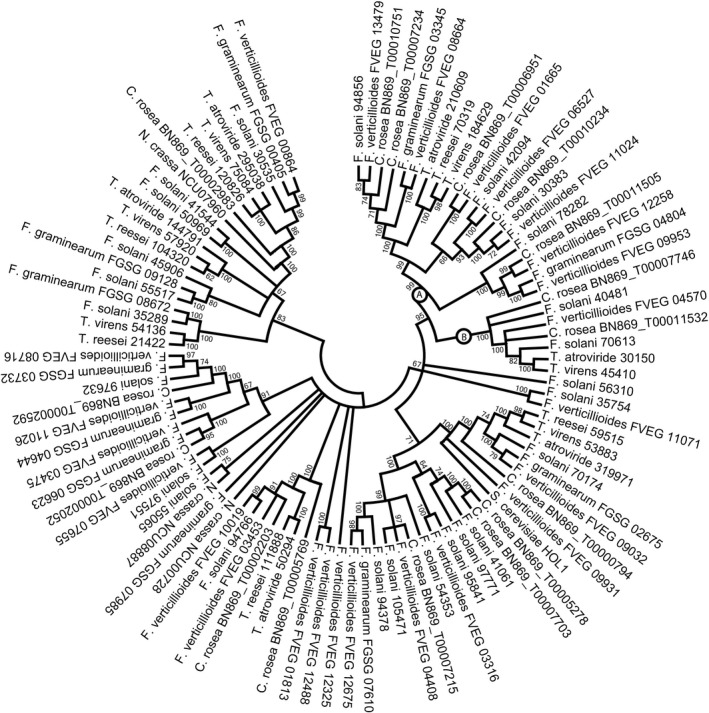
Phylogenetic relationships of MFS transporter family 2.A.1.2.33 among Hypocreales. Predicted amino acid sequences of MFS transporters were aligned by MUSCLE and used to construct a phylogenetic tree using the neighbour‐joining method in the MEGA6 software package. Bootstrap support (≥60%) values from 500 iterations are associated with nodes. Species name and protein ID are given for each MFS transporter

The phylogenetic tree of the MFS 2.A.1.2.33 gene family displayed higher resolution of deeper branches compared with the MFS 2.A.1.3.65 family (Figure 3). However, only two cases of orthologs shared between the seven hypocrealean species were identified (*C. rosea* BN869_T00002983 and BN869_T00000794), indicative of high rates of gene gain and loss. The *C. rosea* MFS464 transporter was not orthologous to the type transporter of the MFS 2.A.1.2.33 family, the HOL1 transporter from *Saccharomyces cerevisiae* with unknown function but possibly involved in transport of histidinol and other cations (Wright, Howell, & Gaber, 1996).

### 3.5 Structural divergence of MFS 2.A.1.3.65 paralogs

**Figure 4:**
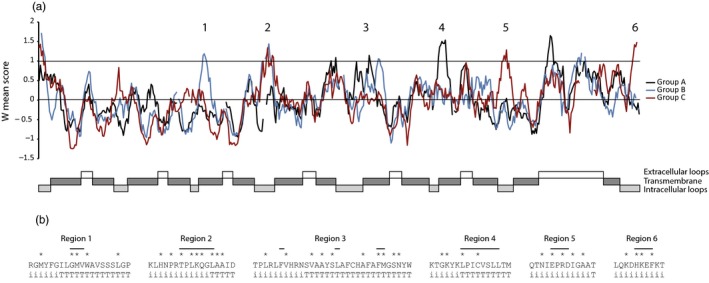
Reverse conservation analysis of family 2.A.1.3.65 MFS transporters. (a) Amino acid conservation was estimated using Rate4Site, based on a MUSCLE alignment of hypocrealean MFS transporters, and plotted as W mean scores (based on S scores for individual positions) in arbitrary units. The black, blue and red lines represent the A, B and C clades indicated in Figure 2, respectively. Regions with signs of functional divergence (W ≥ 1 in one group and W < 0.5 in another group) are indicated. Transmembrane helices and loop structures were predicted with HMMTOP and displayed. (b) Amino acid sequences of regions (indicated by horizontal lines) with signs of functional divergence. Positions with high amino acid diversity (S score ≥1) are indicated by asterisks. Amino acid positions forming parts of transmembrane helices are indicated by T, while positions forming parts of intracellular loops are indicated by i

Alignments and RCA analyses were applied to reveal conserved and variable regions between three MFS 2.A.1.3.65 phylogenetic groups with ≥60% bootstrap support (here referred to as clades A, B and C, Figure 2). Ten regions with high amino acid diversity (W ≥ 1) were identified in the MFS 2.A.1.3.65 alignment (Figure 4). Six of these regions displayed signs of functional divergence, defined as high variation (W ≥ 1) in one group in combination with low variation (W < 0.5) in another group and were labelled 1 through 6 (Figure 4).

Five of these regions (1–5) were predicted to be associated with loops that connect transmembrane helices on the inside of the cell and the beginning of the transmembrane helices themselves (Figure 4). Region 6 was predicted to be part of the C‐terminal, internal loop. Hence, all six regions were predicted to form parts of the entry of the transport tunnel, likely influencing substrate specificity.

### 3.6 Structural divergence of MFS 2.A.1.2.33 paralogs

**Figure 5:**
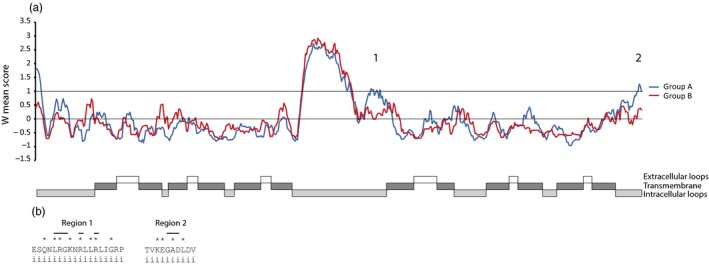
Reverse conservation analysis of family 2.A.1.2.33 MFS transporters. (a) Amino acid conservation was estimated using Rate4Site, based on a MUSCLE alignment of hypocrealean MFS transporters, and plotted as W mean scores (based on S scores for individual positions) in arbitrary units. The blue and red lines represent the A and B clades indicated in Figure 3, respectively. Regions with signs of functional divergence (W ≥ 1 in one group and W < 0.5 in another group) are indicated. Transmembrane helices and loop structures were predicted with HMMTOP and displayed. (b) Amino acid sequences of regions (indicated by horizontal lines) with signs of functional divergence. Positions with high amino acid diversity (S score ≥ 1) are indicated by asterisks. Amino acid positions forming parts of intracellular loops are indicated by i

Four regions with high amino acid diversity (W ≥ 1) were identified by RCA between two MFS 2.A.1.2.33 phylogenetic groups (clades A and B in Figure 3). Only two of these regions displayed signs of functional divergence (W ≥ 1 in group A and W < 0.5 in group B) and were labelled 1 and 2 (Figure 5). Region 1 was located in the end of a large, highly variable internal loop region, while region 2 was located in a C‐terminal internal loop (Figure 5).

### 3.7 Mycotoxin‐ and fungicide‐induced gene expression in *C. rosea*

As many differentially expressed genes belonged to expanded gene families predicted to be involved in drug resistance/secondary metabolite transport, we analysed gene expression of eight genes used for RNA‐seq validation (Figure 1) in *C. rosea* during exposure to the *Fusarium* mycotoxin zearalenone (with antifungal properties [Utermark & Karlovsky, 2007]) and to four fungicides with different mode of action. The ABC transporter gene *abcG18* was induced 11,152‐fold after exposure to zearalenone for 2 hr, while the predicted FAD‐dependent oxidoreductase gene *fdo1* was induced 2.7‐fold by zearalenone (Table 3). The ABC transporter gene *abcC8*, the MFS transporter gene *mfs602* and *fdo1* were induced during exposure to fungicides boscalid and mefenoxam, while *mfs602* and *fdo1* were additionally induced by iprodione (Table 3).

**Table 3:** Expression^a^ of *Clonostachys rosea* genes

Treatment	Genes							
	*mfs293*	*mfs249*	*abcC8*	*mfs602*	*mfs464*	*fdo1*	*ptr1*	*abcG18*
Zearalenone	3.8	ND	0.8	2.4	D	2.7*	18.7	11152.3*
Zearalenone control	1.3	ND	1.0	1.0	ND	1.1	5.5	1.0
Iprodione	ND	ND	1.0	2.0*	ND	7.4*	4.3	ND
Boscalid	ND	ND	3.7*	4.8*	ND	4.2*	1.4	ND
Mefenoxam	ND	ND	0.4*	10.9*	ND	89.3*	4.6	D
Azoxystrobin	ND	ND	1.0	2.2	ND	1.9	ND	ND
Fungicide control	ND	ND	1.1	0.8	ND	1.4	1.1	ND

D, transcripts were detected in ≥3 biological replicates; ND, transcripts were not detected in ≥3 biological replicates.

a Relative gene expression in relation with the appropriate control treatment. An asterisk indicates a significant (*p* ≤ .05) difference compared with the appropriate control treatment as determined by *t* test.

### 3.8 Phenotypic analyses of gene deletion strains in *C. rosea*

*Clonostachys rosea* gene deletion mutants were generated by replacing *mfs602*,* mfs464*,* fdo1* and *cyp1* with the hygromycin selection cassette (hygB) by ATMT. Successful gene replacements in mitotically stable mutants were confirmed by PCR using primers located within the hygB cassette in combination with primers located upstream and downstream of the constructs (Appendix S2) as described previously (Dubey, Broberg, Jensen et al., 2013; Dubey et al., 2012, 2014, 2016). The expected size of PCR fragments were amplified in selected *mfs602*,* mfs464*,* fdo1* and *cyp1* mutant strains, while no amplification was observed in WT (Appendix S2). Furthermore, RT‐PCR experiments using primers specific to *mfs602*,* mfs464*,* fdo1* and *cyp1* demonstrated the complete loss of the respective transcripts in each mutant (Appendix S2).

**Figure 6:**
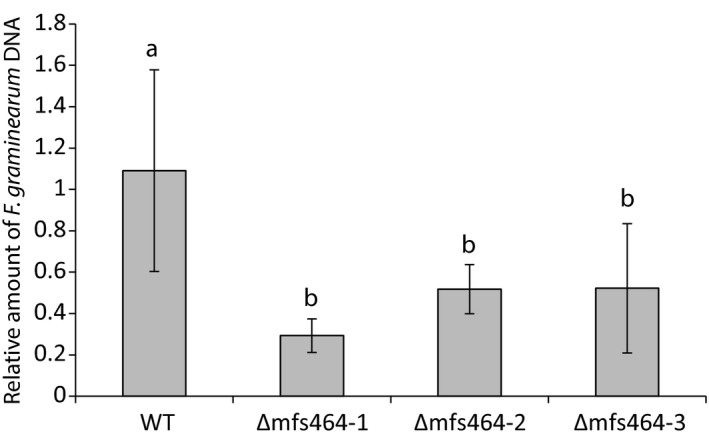
Assay of in vitro antagonism of *C. rosea* WT and ∆*mfs464* mutants. *Clonostachys rosea* WT and three independent Δ*mfs464* strains were inoculated in liquid VM media together with *F. graminearum*. Fungal biomass was estimated after 3 days of interaction by quantifying *C. rosea* and *F. graminearum* DNA concentrations using qPCR. Error bars represent standard deviation based on five biological replicates. Different letters indicate statistically significant differences (*p* ≤ .05) based on the Fisher method

When *C. rosea* WT or the ∆*mfs464* mutants were co‐inoculated with *F. graminearum* in liquid VM with 0.3% glucose, there was a significant (*P* = .001) 2.1–3.7‐fold reduction in *F. graminearum* biomass (measured as *F. graminearum* DNA/*C. rosea* DNA ratio) after 3 days of interaction (Figure 6). No reductions in *F. graminearum* biomass were observed for the ∆*mfs602*, ∆*fdo1* or ∆*cyp1* mutants. No additional phenotypic effects in any of the four *C. rosea* gene deletion mutants were observed for any of the studied phenotypes, including tolerance to mycotoxins, fungicides, chemical agents for cell wall, osmotic or oxidative stress, or ion transport/tolerance, nutrient deficiencies, or in vitro dual culture interaction, or biocontrol of fusarium foot rot on barley (data not shown).

## 4 DISCUSSION

Necrotrophic mycoparasites such as *C. rosea* and *Trichoderma* spp. are assumed to have broad host ranges with little specificity. Evidence to support this view comes from the fact that single species, and sometimes single strains, are efficient biocontrol agents against a range of different plant pathogenic fungi (Jensen et al., 2007), and sometimes even against oomycete and plasmodiophorid pathogens (Lahlali & Peng, 2014; Moller, Jensen, Andersen, Stryhnz, & Hockenhull, 2003). Screening of 75 different species of *Trichoderma* also showed that all possessed mycoparasitic activity against the plant pathogenic species *A. alternata*,* B. cinerea* and *S. sclerotiorum* (Druzhinina et al., 2011). Therefore, it is intriguing to observe that *C. rosea* reacts with both common and specific gene expression during interspecific interactions with *B. cinerea* and *F. graminearum*, suggesting that *C. rosea* can distinguish between fungal prey species and modulate its responses accordingly.

A comparative transcriptomic study of *T. atroviride*,* T. reesei* and *T. virens* during interaction with *Rhizoctonia solani* (Atanasova, Le Crom et al., 2013) revealed both common and specific responses between the three species. The common response included induction of ABC and MFS transporters, proteases and heat shock proteins. *Trichoderma atroviride* mycoparasitism was characterized by induced genes involved in biosynthesis of secondary metabolites and fungal cell wall degradation, but also small, secreted cysteine‐rich proteins, while the predatory mycoparasitism of *T. virens* was associated with induction of genes involved in gliotoxin biosynthesis (Atanasova, Le Crom et al., 2013). The weak mycoparasite and wood‐degrading *T. reesei* induced cellulase and hemicellulase genes.

A comparative genome analysis identified high numbers of genes encoding ABC and MFS transporters, PKS, NRPS, CYP and PL1 genes, and a low number of chitinases, in the *C. rosea* genome (Karlsson et al., 2015), suggesting that their gene products may be involved in ecological niche adaptation (Wapinski et al., 2007) including biotic interactions, nutrient acquisition and stress modulation. It is therefore notable that members from all of these gene families are also induced during interspecific fungal interactions, indicating that mycoparasitism is the main driver for shaping the genome content in *C. rosea*. The common response in *C. rosea* against both fungal preys included sugar and small organic compound transporters that may be involved in nutrient uptake. The specific responses towards the two different fungal preys were dominated by genes predicted to be involved in membrane transport, biosynthesis of secondary metabolites and carbohydrate degradation, which fits well with our current view that degradation of the cell wall of the fungal prey, production of toxic metabolites for poisoning of the fungal prey and the ability to tolerate the counterattack by the fungal prey (by means of toxic metabolites and reactive oxygen species) are key mechanisms for a successful necrotrophic mycoparasite (Druzhinina et al., 2011).

The *chiC1* chitinase gene was previously shown to be induced by chitin (Tzelepis et al., 2015) and is predicted to encode a killer toxin‐like chitinase that permeabilize the cell wall of antagonistic species to facilitate entry of toxic metabolites (Karlsson & Stenlid, 2008; Seidl, Huemer, Seiboth, & Kubicek, 2005). This function was previously confirmed for another killer toxin‐like chitinase in *C. rosea*; deletion of the *chiC2* gene resulted in lower growth‐inhibiting activity of culture filtrates against *B. cinerea* and *R. solani*, but notably, not against *F. graminearum* (Tzelepis et al., 2015). This may be significant as the 50‐fold induction of *chiC1* against *F. graminearum*, but not against *B. cinerea*, may suggest adaptation of killer toxin‐like paralogs towards different fungal preys (driven by differences in cell wall composition, presence of chitinase inhibitors etc.). Differential regulation of killer toxin‐like paralogs was also reported from *T. atroviride* (Gruber, Vaaje‐Kolstad et al., 2011) and *T. virens* (Gruber, Kubicek, & Seidl‐Seiboth, 2011), and the saprotrophic *Aspergillus nidulans* (Tzelepis, Melin, Stenlid, Jensen, & Karlsson, 2014) and *N. crassa* (Tzelepis et al., 2012). The killer toxin‐like function was also confirmed in a nonmycoparasitic species; deletion of the *chiC2‐2* gene in *A. nidulans* resulted in reduced growth‐inhibiting activity of culture filtrates (Tzelepis et al., 2014).

*Clonostachys rosea* is known to produce a limited number of secondary metabolites with biotic activity; peptaibols with toxicity against *S. sclerotiorum* (Rodriguez et al., 2011), polyketides with antibacterial activity (Zhai et al., 2016), glisoprenin inhibitors of appressorium formation (Thines, Eilbert, Anke, & Sterner, 1998) and nematicidal epipolysulfanyldioxopiperazines (Dong, He, Shen, & Zhang, 2005). However, it is not possible at this stage to connect the secondary metabolite biosynthetic genes induced against *B. cinerea* or *F. graminearum* with any of these compounds. The *pks9* gene induced against *B. cinerea* is predicted to encode a reduced lovastatin diketide polyketide, and other PKS genes of the same type were induced during mycoparasitism in both *T. atroviride* (Atanasova, Le Crom et al., 2013) and *T. reesei* (Atanasova, Knox, Kubicek, Druzhinina, & Baker, 2013). Deletion of the nonreducing type *pks4* gene in *T. reesei* resulted in loss of pigmentation and mutants that were more sensitive to toxic metabolites produced by the fungal prey (Atanasova, Knox, Kubicek et al., 2013). Induction of genes with similarity to isotrichodermin C‐15 hydroxylase (*cyp1*) and norsolorinic acid reductase in *C. rosea* during interaction with *F. graminearum* may indicate production of hitherto unknown secondary metabolites, and similar genes were reported to be induced during mycoparasitism in *T*. cf. *harzianum* (Steindorff et al., 2012, 2014; Vieira et al., 2013).

Interpretation of the induction of *C. rosea* ABC and MFS transporters during interaction with *B. cinerea* and *F. graminearum* is made difficult by the fact that both groups of membrane transporters exists as large gene families with a complex phylogenetic substructure that typically reflects their involvement in a wide range of biological functions, including basic metabolism and cellular homoeostasis (Kovalchuk & Driessen, 2010). The ABC transporter gene family in *C. rosea* has been studied in detail (Karlsson et al., 2015), where ABC transporter group B (multidrug resistance proteins) and G (pleiotropic drug resistance proteins) were shown to evolve under selection for increased gene copy numbers. The *abcG18* gene, induced against *F. graminearum* and during exposure to the *Fusarium* mycotoxin zearalenone, indeed belongs to group G, suggesting a role of the ABCG18 protein in protecting *C. rosea* against toxic metabolites produced by fungal prey (at least by *Fusarium* spp.). Three other ABC transporter genes from group G (*abcG5*,* abcG8* and *abcG29*) and one gene from group B (*abcB26*) were also reported to be induced in *C. rosea* during exposure to ZEA (Karlsson et al., 2015; Kosawang, Karlsson, Jensen, Dilokpimol, & Collinge, 2014), and both ABCG5 and ABCG29 were later shown to be involved in biocontrol of fusarium foot rot disease (Dubey et al., 2014, 2016). The *C. rosea abcB1*,* abcB4*,* abcB18*,* abcB20* and *abcB26* group B and the *abcG8* and *abcG25* group G ABC transporter genes were also reported to be induced by exposure to bacterial metabolites (Kamou et al., 2016; Karlsson et al., 2015), suggesting that *C. rosea* needs to defend itself not only to metabolites from the fungal prey but also to antibiosis from the surrounding microflora in the rhizosphere. The *abcC8* gene belongs to the multidrug resistance‐associated proteins, subgroup C‐V (Karlsson et al., 2015) and the induction during interaction with *B. cinerea* and during exposure to fungicides boscalid and mefenoxam may suggest an involvement in cellular protection against exogenous toxic metabolites and xenobiotics.

The MFS transporter gene family on the other hand is not studied to any detail in *C. rosea*, or in any other fungus. Our comprehensive evolutionary analysis of the MFS transporter gene family in hypocrealean fungi revealed selection for increased gene copy numbers in families related with drug resistance/secondary metabolite transport, small organic compound transport and carbohydrate transport in *C. rosea*. MFS transporter genes induced during interactions with *B. cinerea* and *F. graminearum* almost exclusively belonged to these expanded MFS families, indicating that efflux‐mediated protection against exogenous or endogenous secondary metabolites and nutrient uptake are important components of the mycoparasitic attack in *C. rosea*. MFS transporters were reported to be induced in several other mycoparasitic species, including the closely related *C. chloroleuca* (Moreira, Abreu, Carvalho, Schroers, & Pfenning, 2016; Sun, Sun, & Li, 2015), *Trichoderma* spp. (Atanasova, Le Crom et al., 2013; Seidl et al., 2009; Steindorff et al., 2014), *Tolypocladium ophioglossoides* (Quandt, Di, Elser, Jaiswal, & Spatafora, 2016), *Escovopsis weberi* (de Man et al., 2016), *Pythium oligandrum* (Horner, Grenville‐Briggs, & Van West, 2012) and *Ampelomyces quisqualis* (Siozios et al., 2015), but without proper identification of family classification it is difficult to speculate about their exact biological role in these species.

Although gene expression fold‐change induction not necessarily corresponds to the importance of a particular gene product, *mfs602* and *mfs464* stood out in among the MFS transporters with a ≥693‐fold induction during interaction with *F. graminearum*, but not during interaction with *B. cinerea*. This substantial induction, coupled with the fact that both genes belongs to gene families evolving under selection for increased paralog numbers (2.A.1.3.65 and 2.A.1.2.33), indicates that the encoded transporter proteins perform important functions in the mycoparasitic attack of *F. graminearum* by *C. rosea*. The phylogenetic structure of both families shows that they evolve by a rapid birth‐and‐death process followed by sequence diversification, indicating that functional diversification between paralogs is the driving force for the observed gene copy number increase, rather than selection for increased protein amount (where sequence conservation between paralogs are expected). MFS602 belong to family 2.A.1.3.65 predicted to be involved in drug resistance, and our analysis of structural divergence showed that loops forming the entry to the transport channel are the targets of the diversification between paralogs, indicating that selection for substrate specificity has been important for the evolution of MFS family 2.A.1.3.65 members in hypocrealean fungi. The MFS 2.A.1.3.65 family also evolve under different evolutionary trajectories in *Trichoderma* spp., with gene gains in *T. atroviride*, random genetic drift in *T. virens* and gene losses in *T. reesei*, possibly reflecting its current life style transition to a wood‐degrading saprotroph from a mycoparasitic ancestor (Kubicek et al., 2011). The only characterized MFS transporter in *Trichoderma* spp., MFS1 in *T*. cf. *harzianum*, also belongs to family 2.A.1.3.65 and was indeed shown to transport the *Trichoderma* spp. metabolite trichodermin and consequently affect in vitro antagonism (Liu et al., 2012). MFS1 was also shown to transport xenobiotics such as iprodione and a range of azole‐type fungicides. This dual function fits well with the gene expression profile of *mfs602* that was induced during interaction with *F. graminearum* but also during exposure to the fungicides iprodione, boscalid and fenhexamid. However, deletion of the *mfs602* gene in *C. rosea* did not result in any measurable phenotype. This may not be unexpected, given the large number of similar paralogs present in *C. rosea* that can mask any potential effect by partially overlapping functions, which is previously reported for other large gene families such as chitinases and hydrophobins (Alcazar‐Fuoli et al., 2011; Mosbach, Leroch, Mendgen, & Hahn, 2011).

The function of MFS464 is more difficult to speculate about, as no member of MFS family 2.A.1.2.33 is characterized to any detail. Even the type transporter of the family, HOL1 from *S. cerevisiae*, was only shown to transport histidinol after random mutagenesis, which not necessarily provides evidence for its true biological function (Wright et al., 1996). As with family 2.A.1.3.65, the phylogenetic analysis of family 2.A.1.2.33 suggests functional diversification between paralogs, but the structure of these transporters was very different from 2.A.1.3.65 members with one large internal loop. Part of this loop was highly variable in sequence between subgroups, indicating low selective constraints, but another part of this loop displayed a signature of functional diversification. We may speculate that this large, variable internal loop can interact with other proteins, perhaps forming a signalling complex, but this remains to be investigated. Deletion of *mfs464* in *C. rosea* resulted in mutants that displayed increased growth inhibitory activity against *F. graminearum* when co‐inoculated in liquid medium, providing evidence for a function of MFS464 in interspecific fungal interactions, although the exact nature of this effect remains to be investigated.

In summary, we show that the necrotrophic mycoparasite *C. rosea* can distinguish between fungal prey species and modulate its transcriptomic responses accordingly. Genes predicted to encode membrane transporters involved in drug resistance/secondary metabolite transport and biosynthesis of PKS and NRPS secondary metabolites forms a large part of the interaction transcriptomes, emphasizing the importance of secondary metabolites in mycoparasitic interactions. The overlap between genes induced during the mycoparasitic attack and genes/gene families evolving under diversifying selection is equivalent to the effector concept in parasite–host interactions where the co‐evolutionary arms race results in rapidly evolving parasite effectors and host receptors. Identification of mechanisms resulting in successful biocontrol of plant pathogenic fungi is important for knowledge‐based improvements of biocontrol efficacy in agricultural production systems.

## CONFLICT OF INTEREST

None declared.

## DATA ARCHIVING STATEMENT

The transcriptome sequence data generated and analysed in this work are deposited in the short read archive (SRA) at NCBI, with accession numbers SRX3389264 ‐ SRX3389269.
