# Supplementary material for: The mycoparasitic fungus Clonostachys rosea responds with both common and specific gene expression during interspecific interactions with fungal prey
Source: Evol Appl. 2018 Mar 14;11(6):931–49. doi: 10.1111/eva.12609 (PMC5999205; doi:10.1111/eva.12609)
Supplement: Supplementary file 1 [file EVA-11-931-s001.docx]

**Appendix A:** List of primers used in this study.

| Name | Target gene | Sequence (5´ → 3´) | Tm (°C) | Amplicon length (bp) |
| --- | --- | --- | --- | --- |
| abcC8F | *abcC8* | GCG CTG CCA CTC CCG TTC T | 60 | 172 |
| abcC8R |  | CAA GTC GCC GCA GCA AGG T | 61 |  |
| abcG18F | *abcG18* | GCC CAA TAT CGT GCC CAA GTC A | 61 | 146 |
| abcG18R |  | GAA GCG CCA GCA ATC AAC ATC TC | 60 |  |
| cyp1F | *cyp1* | CAC GGA ACT GCA TCG GGA AAA A | 59 | 113 |
| cyp1R |  | TTCACGTTTAGTTTGCCCTTCTGC | 60 |  |
| fdo1F | *fdo1* | GCC CTG GGT CTA GCT CCG TGT T | 60 | 190 |
| fdo1R |  | TCC GCA TAT CCG CCG AAG AAT | 60 |  |
| mfs249F | *mfs249* | AGA TAT GAT ACC CCC GAT GCC AGA T | 60 | 151 |
| mfs249R |  | TCC GTT GCG AGA CCG ATG TTT C | 61 |  |
| mfs293F | *mfs293* | CGC CCC GAT GCT CAT TGT TAC TAC | 61 | 171 |
| mfs293R |  | GGA AGC AGG GGC GAT GTT GTT A | 60 |  |
| mfs464F | *mfs464* | GCC CTT TTC CGC TTG CTA TGG | 60 | 172 |
| mfs464R |  | ACC GCG CTC GTG AGT GAA GTA AAT | 60 |  |
| mfs602F | *mfs602* | GCG CTG TGT ACT ACC TTG CCA TCT G | 61 | 196 |
| mfs602R |  | ATT CCG CCG CCC ACA CTA CAT A | 60 |  |
| ptr1F | *ptr1* | CCT CGC CTA CGC TCT CCC TCT TA | 60 | 159 |
| ptr1R |  | ACC TTG GCG TTT CCG TTC TCG | 60 |  |
| 6455 ups F | *mfs602* upstream | ^1^GGGG ACA ACT TTG TAT AGA AAA GTT G GGG CTG TGA AGG GCT CTA TGA AGT | 59 | 905 |
| 6455 ups R |  | ^1^GGGG AC TGC TTT TTT GTA CAA ACT TG TGT GGA GGA AGG CAT CGT CGT | 59 |  |
| 6455 ds F | *mfs602* downstream | ^1^GGGG ACA GCT TTC TTG TAC AAA GTG G CCA GAA GAA CCA GGC TCC AGA AA | 58 | 1033 |
| 6455 ds R |  | ^1^GGGG AC AAC TTT GTA TAA TAA AGT TG TCA ACC CGC GAC CGA TTC C | 60 |  |
| 6455 ko F | *mfs602* | CAC GCT ATC CAG ATC CTT ATG AGA A | 54 | Not applicable |
| 6455 ko R |  | CTC TCG GCA GGC TCT CCA A | 55 |  |
| Hyg F | *hph* | GCG CGC AAT TAA CCC TCA C |  |  |
| Hyg R |  | GAA TTG CGC GTA CAG AAC TCC |  |  |
| 7234 ups F | *mfs464* upstream | ^1^GGGG ACA ACT TTG TAT AGA AAA GTT G ACG CAC CGG TCA TTG TTG G | 56 | 818 |
| 7234 ups R |  | ^1^GGGG AC TGC TTT TTT GTA CAA ACT TG GGA CCG GAG CCC TGT TCG | 57 |  |
| 7234 ds F | *mfs464* downstream | ^1^GGGG ACA GCT TTC TTG TAC AAA GTG G TGC AGC ATT GAG GGA GGA CA | 58 | 1033 |
| 7234 ds R |  | ^1^GGGG AC AAC TTT GTA TAA TAA AGT TG AAG TGA CGG CAG ATG ATA GGT TCC | 60 |  |
| 7234 ko F | *mfs464* | CAA GGG TAG CCT GTT GAT TAG TGG |  | Not applicable |
| 7234 ko R |  | AAA ATC TCG GTC AGC CAT CCA T |  |  |
| 606 ups F | *fdo1* upstream | ^1^GGGG ACA ACT TTG TAT AGA AAA GTT G CCG ACG AGG CCC GAG TGA | 59 | 918 |
| 606 ups R |  | ^1^GGGG AC TGC TTT TTT GTA CAA ACT TG CTG TCG AGA AGC CAC CTG AGC A | 59 |  |
| 606 ds F | *fdo1* downstream | ^1^GGGG ACA GCT TTC TTG TAC AAA GTG G AGA GTG GAG CTG GGA AGG AAT GA | 58 | 958 |
| 606 ds R |  | ^1^GGGG AC AAC TTT GTA TAA TAA AGT TG CCT GCA GCA TCG GGG TTT TAT | 58 |  |
| 606 ko F | *fdo1* | GAG CCG GCA TGG TCC TTG TG |  | Not applicable |
| 606 ko R |  | GAA ATG GGA AGA TCA CCG AAA TGT |  |  |
| 852 ups F | *cyp1* upstream | ^1^GGGG ACA ACT TTG TAT AGA AAA GTT G GCT GCT ATT CAC TGC CTC ATC ATT | 57 | 963 |
| 8520 ups R |  | ^1^GGGG AC TGC TTT TTT GTA CAA ACT TG AGA TAC TCG CGG TGG GAC TCA T | 57 |  |
| 852 ds F | *cyp1* downstream | ^1^GGGG ACA GCT TTC TTG TAC AAA GTG G TGT GGC AGA AGG GCA AAC TAA AC | 57 | 937 |
| 852 ds R |  | ^1^GGGG AC AAC TTT GTA TAA TAA AGT TG CGA CGT CGA CCT TGA TGA GAA CC | 59 |  |
| 852 ko F | *cyp1* | TCG CTG TTC AAA TCA TTC CTT CAA | 58 | Not applicable |
| 852 ko R |  | CAG CAG TCA TTG GCA ACG GAT T | 59 |  |

^1^attB and attBr sequences for gateway cloning are underlined.

Abbreviation used for enzyme-encoding genes: *hph* = Hygromycin B phosphotransferase.
