# Supplementary material for: The mycoparasitic fungus Clonostachys rosea responds with both common and specific gene expression during interspecific interactions with fungal prey
Source: Evol Appl. 2018 Mar 14;11(6):931–49. doi: 10.1111/eva.12609 (PMC5999205; doi:10.1111/eva.12609)
Supplement: Supplementary file 2 [file EVA-11-931-s002.pdf]

Appendix B: Schematic representation of gene deletion cassettes and mutant validation.

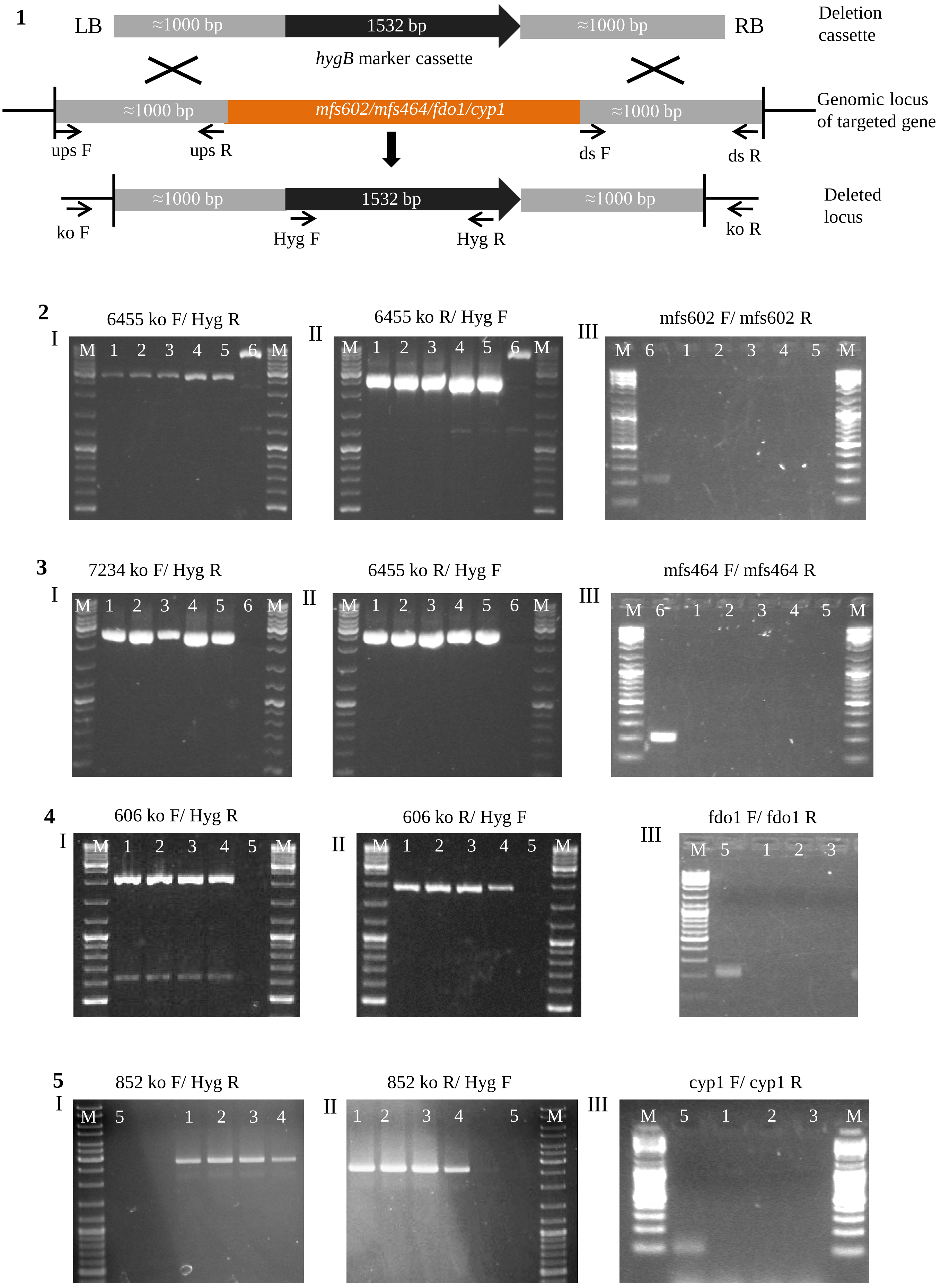

Appendix B: Schematic representation of gene deletion cassettes and mutant validation.

1: Organisation of *mfs602, mfs464, fdo1 or cyp1* locus in WT and mutant strains of *C. rosea*. The coding region of respective gene was replaced by hygB cassette by homologous recombination resulting in generation of deletion strains. The arrow heads indicate the location of primers used to construct the deletion cassette and analysis of mutants using PCR. Abbreviations: LB, left border; RB, right border.

2: PCR verification of Δ*mfs602* using primers located in the *hph* gene (Hyg F / Hyg R) in combination with primers located upstream and downstream from the deletion cassette (6455 ko F / 6455 ko R). A PCR product of (I) ~2.7 kb using primers 6455 ko F / Hyg R, and (II) 6455 ko R / Hyg F were expected from a correct gene replacement. (III) RT-PCR analysis of *mfs602* gene expression in WT and deletion strains using *mfs602* specific primers. A PCR product of 196 bp was expected from WT. M, gene ruler DNA ladder mix; 1-5, independent Δ*mfs602* mutants; 6, WT strain. Primer combinations used for PCR and RT-PCR are given above the images.

3: PCR verification of Δ*mfs464* using primers located in the *hph* gene (Hyg F / Hyg R) in combination with primers located upstream and downstream from the deletion cassette (7234 ko F / 7234 ko R). A PCR product of (I) ~2.5 kb using primers 7234 ko F / Hyg R, and (II) 7234 ko R / Hyg F were expected from a correct gene replacement. (III) RT-PCR analysis of *mfs464* gene expression in WT and deletion strains using *mfs464* specific primers. A PCR product of 172 bp was expected from WT. M, gene ruler DNA ladder mix; 1-5, independent Δ*mfs464* mutants; 6, WT strain. Primer combinations used for PCR and RT-PCR are given above the images.

4: PCR verification of Δ*fdo1* using primers located in the *hph* gene (Hyg F / Hyg R) in combination with primers located upstream and downstream from the deletion cassette (606 ko F / 606 ko R). A PCR product of (I) ~2.5 kb using primers 606 ko F / Hyg R, and (II) 606 ko R / Hyg F were expected from a correct gene replacement. (III) RT-PCR analysis of *fdo1* gene expression in WT and deletion strains using *fdo1* specific primers. A PCR product of 190 bp was expected from WT. M, gene ruler DNA ladder mix; 1-4, independent Δ*fdo1* mutants; 5, WT strain. Primer combinations used for PCR and RT-PCR are given above the images.

5: PCR verification of Δ*cyp1* using primers located in the *hph* gene (Hyg F / Hyg R) in combination with primers located upstream and downstream from the deletion cassette (852 ko F / 852 ko R). A PCR products of (I) ~2.8 kb using primers 852 ko F / Hyg R, and (II) 852 ko R / Hyg F were expected from a correct gene replacement. (III) RT-PCR analysis of *cyp1* gene expression in WT and deletion strains using *cyp1* specific primers. A PCR product of 113 bp was expected from WT. M, gene ruler DNA ladder mix; 1-4, independent Δ*cyp1* mutants; 5, WT strain.

Primer combinations used for PCR and RT-PCR are given above the images.
